# Evaluation of a Physician Peer-Benchmarking Intervention for Practice Variability and Costs for Endovenous Thermal Ablation

**DOI:** 10.1001/jamanetworkopen.2021.37515

**Published:** 2021-12-14

**Authors:** David P. Stonko, Chen Dun, Christi Walsh, Marlin Shul, John Blebea, Edward M. Boyle, Martin A. Makary, Caitlin W. Hicks

**Affiliations:** 1 Department of Surgery, The Johns Hopkins Hospital, Baltimore, Maryland; 2 R. Adams Cowley Shock Trauma Center, Baltimore, Maryland; 3 Center for Vein Restoration, Dothan, Alabama; 4 Central Michigan University College of Medicine, Mount Pleasant; 5 Inovia Vein Specialty Centers, Bend, Oregon; 6 Department of Health Policy & Management, Johns Hopkins Bloomberg School of Public Health, Baltimore, Maryland; 7 Division of Vascular Surgery and Endovascular Therapy, Department of Surgery, The Johns Hopkins Hospital, Baltimore, Maryland

## Abstract

**Importance:**

The frequency of use of endovenous thermal ablation (EVTA) to treat chronic venous insufficiency has increased rapidly in the US. Wide variability in EVTA use among physicians has been documented, and standard EVTA rates were defined in the 2017 Medicare database.

**Objective:**

To assess whether providing individualized physician performance reports is associated with reduced variability in EVTA use and cost savings.

**Design, Setting, and Participants:**

This prospective quality improvement study used data from all US Medicare patients aged 18 years or older who underwent at least 1 EVTA between January 1, 2017, and December 31, 2017, and between January 1, 2019, and December 31, 2019. All US physicians who performed at least 11 EVTAs yearly for Medicare patients in 2017 and 2019 were included in the assessment.

**Intervention:**

A performance report comprising individual physician EVTA use per patient with peer-benchmarking data was distributed to all physicians in November 2018.

**Main Outcomes and Measures:**

The mean number of EVTAs performed per patient was calculated for each physician. Physicians who performed 3.4 or more EVTA procedures per patient per year were considered outliers. The change in the number of procedures from 2017 to 2019 was analyzed overall and by inlier and outlier status. An economic analysis was also performed to estimate the cost savings associated with the intervention.

**Results:**

A total of 188 976 patients (102 222 in 2017 and 86 754 in 2019) who had an EVTA performed by 1558 physicians were included in the analysis. The median patient age was 72.2 years (IQR, 67.9-77.8 years); 67.3% of patients were female, and 84.9% were White. Among all physicians, the mean (SD) number of EVTAs per patient decreased from 2017 to 2019 (1.97 [0.85] vs 1.89 [0.77]; *P* < .001). There was a modest decrease in the mean number of EVTAs per patient among inlier physicians (1.83 [0.57] vs 1.78 [0.55]; *P* < .001) and a more substantial decrease among outlier physicians (4.40 [1.01] vs 3.67 [1.41] ; *P* < .001). Outliers in 2017 consisted of 90 physicians, of whom 71 (78.9%) reduced their EVTA use after the intervention. The number of EVTAs per patient decreased by a mean (SD) of 0.09 (0.46) procedures overall (median, 0.10 procedures [IQR, −0.10 to 0.30 procedures]; *P* < .001). The estimated cost savings associated with the decrease was $6.3 million in 2019.

**Conclusions and Relevance:**

In this quality improvement study, substantial variability in the number of EVTAs performed per patient was observed across the US. When physicians were provided with a 1-time peer-benchmarked performance report card, the timing of the intervention was associated with a significant decrease in the number of EVTAs performed per patient, particularly among outlier physicians. This quality improvement initiative was associated with reduced variability in EVTA use in the US and a substantial savings for Medicare.

## Key Points

**Question:**

Is provision of individualized peer-benchmarking data on performance of endovenous thermal ablation (EVTA) associated with changes in physicians’ practice patterns or costs?

**Findings:**

In this quality improvement study of 1558 physicians who performed at least 11 EVTAs for a total of 188 976 Medicare patients and were given a performance report of their EVTA use compared with their peers, a significant reduction in the number of EVTAs per patient was found after receipt of the reports, resulting in savings of $6.3 million to Medicare per year.

**Meaning:**

In this quality improvement study, peer benchmarking was associated with a change in practice patterns of EVTA-performing physicians and with substantial Medicare savings.

## Teaser

This quality improvement study uses Medicare claims data to evaluate the association of a peer-benchmarking intervention with physician variability in the use of endovenous thermal ablation (EVTA) and related costs.

## Introduction

Chronic venous insufficiency affects 23% to 33% of individuals aged 18 to 64 years in the US, with the incidence being higher in the aging Medicare population.^1,2,3,4^ The contemporary management of chronic venous insufficiency has shifted from hospital-based open surgical interventions toward minimally invasive procedures,^1,5^ including endovenous thermal ablation (EVTA). Endovenous thermal ablation has been shown to be associated with shortened recovery time, decreased postoperative pain, and improved long-term quality of life, with lower rates of varicose vein recurrence compared with conventional open surgery.^5,6^ Accordingly, the rates of EVTA use in the US have increased consistently over the past 20 years.^7,8^

Given these trends, the American Vein and Lymphatic Society, in conjunction with investigators from The Johns Hopkins School of Medicine, previously set out to assess EVTA use in the US^8^ and identified physician and patient characteristics associated with EVTA practice variation using a physician-developed performance metric and applied the metric to Medicare insurance claim data. In that study, national practice patterns of EVTA use were characterized and physicians performing high numbers of EVTAs per patient were identified. After those data were analyzed in 2017, national and confidential individual physician data were shared with all physicians in the analysis in the form of individualized peer-benchmarked performance reports.

We hypothesized that sharing peer-benchmarked practice data with EVTA-performing physicians would decrease practice variability among physicians who conduct EVTAs at the highest rates. The aim of this study was to assess whether providing these individualized performance reports to all US physicians treating Medicare patients would be associated with reduced variability in EVTA use and reduced costs nationally.

## Methods

### Study Population

This quality improvement study used 100% Medicare fee-for-service carrier claims to identify all Medicare patients aged 18 years or older who underwent at least 1 EVTA between January 1, 2017, and December 31, 2017, and between January 1, 2019, and December 31, 2019, using the *Current Procedural Terminology* (*CPT*) codes for radiofrequency ablation (36475, 36476) and endovenous laser ablation (36478, 36479). This research was approved by the Johns Hopkins Institutional Review Board; the requirement for informed consent was waived because analyzed data did not include patient-identifiable information. This study followed the Standards for Quality Improvement Reporting Excellence (SQUIRE) reporting guidelines for quality improvement studies and adhered to the associated checklist.

### Patient Characteristics

Medicare beneficiaries who underwent EVTA during the study period were identified within the Medicare Master Beneficiary Summary File, from which we obtained the patients’ demographic data.^9^ Patient zip codes were mapped to the Federal Information Processing Standard code. The Federal Information Processing Standard code was subsequently mapped to the core-based statistical area code using a county crosswalk from the National Bureau of Economic Research,^10^ allowing identification of the patients’ metropolitan area type: urban core (50 000 people or more), rural area (10 000-49 999 people), or non–core-based statistical area. We examined each EVTA encounter to determine its indication using diagnosis codes (eTable in the Supplement).

### Physician Population and Characteristics

National Provider Identifier numbers were used to identify the proceduralist for each Medicare claim. Physicians who treated fewer than 11 patients during either 2017 or 2019 were excluded according to our Medicare data use agreement. We subanalyzed physicians in both the 2017 and the 2019 analyses to track changes in physician performance over time. Physician characteristics were derived from Medicare Data on Provider Practice^11^ and the Specialty and Medicare Physician National Downloadable File.^12^ Physician specialty was based on the provider taxonomy code set, which is self-reported by physicians; physicians’ taxonomy does not necessarily correlate with subspecialty board certification.

### Quality Improvement Initiative and Outcomes

In prior work,^8^ physicians who performed an outlier number of mean EVTAs per patient in 2017 were identified. This outlier number was defined as 2 or more SDs above the mean based on consensus with the American Vein and Lymphatic Society, equal to 3.4 or more EVTAs per patient; this value was validated against other statistical cutoffs and held constant during study years. All physicians were provided with an individualized performance report by mail (distributed in November 2018) that showed their EVTA use per patient compared with that of their peers (eFigure 1 in the Supplement). Physician EVTA use was then tracked for the following 12-month period. The primary outcome of our study was the mean number of EVTAs performed per patient by each physician in 2019 compared with 2017, computed by dividing the total number of reimbursed EVTA codes billed for 1 year by a given physician by the number of individual Medicare patients treated with EVTA in the same year.

### Statistical Analysis

We reported characteristics of patients using descriptive and univariate statistics. The mean and median numbers of EVTAs per patient for each physician in 2019 vs 2017 were analyzed and stratified by inlier and outlier status using paired *t* tests and the Mood median test,^13^ respectively. A hierarchical linear regression model was used to assess the association of both patient and physician characteristics with EVTA use. The dependent variable for the model was EVTA use, in which the number of EVTAs was modeled as a continuous variable. The independent variables in the model were patient characteristics (age, sex, race and ethnicity, and procedure indication [first level of hierarchical model]) and physician characteristics (sex, years since medical school graduation, practice region, population density of practice location, primary specialty, and annual volume of patients undergoing venous ablation [second level of hierarchical model]). To estimate the cost savings achieved with the quality improvement initiative, we calculated the product of the mean Medicare payments for EVTA per patient in 2019, total number of patients treated by physicians in both 2017 and 2019, and the change in per-patient EVTA use from 2017 to 2019. *P* values were 2-sided, and statistical significance was set a priori at *P* < .05 for all analyses. Statistical analyses were performed using SAS Enterprise, version 7.1 (SAS Institute Inc).

A sensitivity analysis was also performed. New *CPT* codes for noncompounded foam (36465 and 36466) and cyanoacrylate adhesive (36482 and 36483) were introduced in 2018. To evaluate whether changes observed in use of EVTA in 2019 were associated with introduction of this technology, we performed a sensitivity analysis including the original EVTA *CPT* codes as well as the new chemical ablation and adhesive *CPT* codes to define EVTA use in 2019.

## Results

### Patient Cohort

A total of 188 976 patients (102 222 in 2017 and 86 754 in 2019) underwent EVTA during the study period (Table 1). Overall, the median patient age was 72.2 years (IQR, 67.9-77.8 years); 67.3% of patients were female, and 84.9% were White. The median age was 72.1 years (IQR, 67.9-77.8 years) in 2017 and 72.3 years (IQR, 68.0-77.7 years) in 2019. Among the patients in the 2017 cohort, 67.8% were female, 32.2% were male, 84.9% were White, 44.3% were from the South, and 84.8% were from a metropolitan area. The median number of EVTAs performed per patient was 2 (IQR, 1-3), and most EVTAs (93.6%) were performed because of pain, swelling, or inflammation. The 2019 cohort was clinically similar to the 2017 cohort: 66.7% were female, 33.3% of patients were male, 84.9% were White, and 46.3% were from the South. The median number of EVTAs performed per patient was 2 (IQR, 1-2), and 94.2% of procedures were performed because of pain, swelling, or inflammation.

**Table 1.** Demographic Characteristics of Medicare Patients Who Underwent Endovenous Thermal Ablation in 2017 and 2019

Characteristic	Patients^a^	
	2017 (n = 102 222)	2019 (n = 86 754)
Age, y		
Median (IQR)	72.1 (67.9-77.8)	72.3 (68.0-77.7)
18-64	10 359 (10.1)	7938 (9.2)
65-74	54 662 (53.5)	47 448 (54.7)
75-84	30 061 (29.4)	25 666 (29.6)
85-94	6952 (6.8)	5488 (6.3)
≥95	188 (0.2)	214 (0.3)
Sex		
Female	69 318 (67.8)	57 903 (66.7)
Male	32 904 (32.2)	28 851 (33.3)
Race and ethnicity		
Alaska Native or American Indian	312 (0.3)	273 (0.3)
Asian	1687 (1.7)	1345 (1.6)
Black	6033 (5.9)	5226 (6.0)
Hispanic	4015 (3.9)	3042 (3.5)
White	86 826 (84.9)	73 659 (84.9)
Other or unknown^b^	3349 (3.3)	3209 (3.7)
Region of residence		
Midwest	18 836 (18.4)	15 810 (18.2)
Northeast	15 543 (15.2)	14 178 (16.3)
South	45 239 (44.3)	40 195 (46.3)
West	22 517 (22.0)	16 474 (19.0)
Other	87 (0.1)	97 (0.1)
Population density of residence		
Metropolitan	86 730 (84.8)	73 787 (85.0)
Rural	15 492 (15.2)	12 967 (15.0)
Venous thermal ablations, median (IQR)	2.0 (1.0-3.0)	2.0 (1.0-2.0)
Indication		
Pain, swelling, or inflammation	95 662 (93.6)	81 699 (94.2)
Ulceration	6560 (6.4)	5055 (5.8)

^a^ Data are presented as number (percentage) of patients unless otherwise indicated.

^b^ Other racial and ethnic groups included Native Hawaiian or Other Pacific Islander or no affiliation (replied “don't know” or “refused” to all categories).

### Physician Cohort

A total of 1558 physicians performed more than 10 EVTAs in both 2017 and 2019 (Table 2). Of these physicians, 69 (4.4%) performed 3.4 or more EVTAs per patient in 2019 and were considered outliers in the postintervention period. In 2019, most physicians in both the inlier and the outlier groups were male (1336 [90.0%] in the inlier group and 61 [88.4%] in the outlier group). There was no difference in years since medical school graduation, practice region, population density of practice location, or primary specialty between the inlier and outlier groups (Table 2). The median number of patients treated with EVTA in 2019 was 26 (IQR, 16-45) in the inlier group and 49 (IQR, 30-88) in the outlier group (*P* < .001).

**Table 2.** Characteristics of Physicians Performing EVTA for Medicare Patients

Characteristic	Physicians^a^		*P* value
	Standard use of EVTA (n = 1489)^b^	High use of EVTA (n = 69)^c^	
Sex			
Female	153 (10.3)	8 (11.6)	.73
Male	1336 (90.0)	61 (88.4)	
Time since medical school graduation, y			
Median (IQR)	27.0 (20.0-34.0)	25.0 (19.0-29.0)	.02
0-10	34 (2.9)	0	.09
11-20	346 (23.2)	21 (30.4)	
21-30	562 (37.7)	32 (46.4)	
≥31	532 (35.7)	15 (21.7)	
Unknown	15 (1.0)	1 (1.5)	
Practice region			
Midwest	278 (18.7)	10 (14.5)	.22
Northeast	246 (16.5)	6 (8.7)	
South	687 (45.5)	34 (49.3)	
West	285 (19.1)	19 (27.5)	
Other	2 (0.1)	0	
Population density of practice location			
Metropolitan	1392 (93.5)	66 (95.7)	.62
Rural	97 (6.5)	3 (4.6)	
Primary specialty			
Vascular surgery	475 (31.9)	16 (23.2)	.48
General surgery	233 (15.7)	16 (23.2)	
Cardiothoracic surgery	64 (4.3)	3 (4.4)	
Cardiology	101 (6.8)	5 (7.6)	
Radiology	135 (9.1)	8 (11.6)	
Other	481 (32.3)	21 (30.4)	
Annual volume of patients undergoing venous ablation			
Median (IQR)	26.0 (16.0-45.0)	49.0 (30.0-88.0)	<.001
11-18	494 (33.2)	4 (5.8)	<.001
19-34	465 (31.2)	17 (24.6)	
≥35	530 (35.6)	48 (69.6)	

Abbreviation: EVTA, endovenous thermal ablation.

^a^ Data are presented as number (percentage) of physicians unless otherwise indicated.

^b^ Standard use was fewer than 3.4 EVTAs per patient in 2019.

^c^ High use was 3.4 or more EVTAs per patient in 2019.

### Patient and Physician Factors Associated With EVTA

The number of EVTAs per patient per year was evaluated relative to patient and physician characteristics (Table 3). Compared with the group of patients aged 65 to 74 years, older age was associated with a decreasing number of EVTAs (75-84 years: adjusted difference [AD], −0.07 [95% CI, −0.10 to −0.05]; 85-94 years: AD, −0.25 [95% CI, −0.29 to −0.20]; ≥95 years: AD, −0.45; 95% CI, −0.68 to −0.23; *P* < .001 for all). Female sex (AD, −0.13; 95% CI, −0.15 to −0.10) and Black race (compared with White race: AD, −0.16; 95% CI, −0.22 to −0.11) were also associated with fewer EVTAs per patient, and EVTA performed for ulceration was associated with more EVTAs per patient per year (compared with pain, swelling, or inflammation: AD, 0.47; CI, 0.41-0.53).

**Table 3.** Linear Regression of Factors Associated With Number of Ablations per Year in 2017 and 2019

Characteristic	Unadjusted difference in No. of ablations (95% CI)	*P* value	Adjusted difference in No. of ablations (95% CI)	*P* value
**Patients**				
Age, y				
18-64	−0.08 (−0.13 to −0.03)	.002	−0.04 (−0.08 to 0.01)	.10
65-74	1 [Reference]	NA	1 [Reference]	NA
75-84	−0.11 (−0.14 to −0.08)	<.001	−0.07 (−0.10 to −0.05)	<.001
85-94	−0.27 (−0.32 to −0.21)	<.001	−0.25 (−0.29 to −0.20)	<.001
≥95	−0.28 (−0.54 to −0.01)	.04	−0.45 (−0.68 to −0.23)	<.001
Sex				
Female	−0.10 (−0.13 to −0.07)	<.001	−0.13 (−0.15 to −0.10)	<.001
Male	1 [Reference]	NA	1 [Reference]	NA
Race and ethnicity				
Alaska Native or American Indian	−0.11 (0.35 to 0.14)	.40	0.02 (−0.23 to 0.20)	.87
Asian	0.01 (−0.10 to 0.12)	.89	−0.08 (−0.18 to 0.02)	.10
Black	−0.16 (−0.22 to −0.11)	<.001	−0.16 (−0.22 to −0.11)	<.001
Hispanic	0.12 (0.05 to 0.19)	.009	−0.05 (−0.11 to 0.02)	.17
White	1 [Reference]	NA	1 [Reference]	NA
Other or unknown^a^	0.08 (0.00 to 0.15)	.04	0.01 (−0.07 to 0.05)	.67
Indication				
Pain, swelling, or inflammation	1 [Reference]	NA	1 [Reference]	NA
Ulceration	0.31 (0.25 to 0.37)	<.001	0.47 (0.41 to 0.53)	<.001
**Physicians**				
Sex				
Female	0.07 (0.02 to 0.11)	.01	0.11 (−0.04 to 0.26)	.16
Male	1 [Reference]	NA	1 [Reference]	NA
Time since medical school graduation, y				
0-10	0.19 (0.09 to 0.29)	<.001	0.16 (−0.16 to 0.48)	.33
11-20	0.16 (0.12 to 0.20)	<.001	0.09 (−0.04 to 0.21)	.16
21-30	0.19 (0.16 to 0.22)	<.001	0.03 (−0.07 to 0.14)	.54
≥31	1 [Reference]	NA	1 [Reference]	NA
Practice region				
Midwest	−0.06 (−0.10 to −0.02)	.003	−0.00 (−0.13 to 0.13)	.99
Northeast	−0.25 (−0.29 to −0.20)	<.001	−0.16 (−0.30 to −0.03)	.02
South	1 [Reference]	NA	1 [Reference]	NA
West	0.28 (0.25 to 0.32)	<.001	0.19 (0.07 to 0.31)	.01
Other	−1.05 (−1.62 to −0.47)	.002	−0.58 (−1.88. 0.72)	.38
Population density of practice location				
Metropolitan	1 [Reference]	NA	1 [Reference]	NA
Rural	0.10 (−0.04 to 0.15)	.003	−0.13 (−0.23 to 0.06)	.18
Primary specialty				
Vascular surgery	1 [Reference]	NA	1 [Reference]	NA
General surgery	0.38 (0.34 to 0.42)	<.001	0.39 (0.21 to 0.44)	<.001
Cardiothoracic surgery	0.38 (0.32 to 0.45)	<.001	0.36 (0.13 to 0.60)	.02
Cardiology	−0.06 (−0.13 to 0.00)	.050	0.01 (−0.21 to 0.19)	.92
Radiology	0.50 (0.45 to 0.55)	<.001	0.42 (0.25 to 0.59)	<.001
Other	0.25 (0.21 to 0.28)	<.001	0.27 (0.15 to 0.38)	<.001
Annual volume of patients undergoing venous ablations				
11-18	1 [Reference]	NA	1 [Reference]	NA
19-34	0.27 (0.23 to 0.32)	<.001	0.22 (0.12 to 0.31)	<.001
≥35	0.77 (0.73 to 0.81)	<.001	0.63 (0.54 to 0.73)	<.001

Abbreviation: NA, not applicable.

^a^ Other racial and ethnic groups included Native Hawaiian or Other Pacific Islander or no affiliation (replied “don't know” or “refused” to all categories).

Physicians practicing in the Northeast (compared with those practicing in the South) performed fewer EVTAs per patient (AD, −0.16; 95% CI, −0.30 to −0.03). Physicians with specialties of general surgery (AD, 0.39; 95% CI, 0.21-0.44), cardiothoracic surgery (AD, 0.36; 95% CI, 0.13-0.60), radiology (AD, 0.42; 95% CI, 0.25-0.59), and other (AD, 0.27; 95% CI, 0.15 to 0.38) performed more EVTAs per patient compared with vascular surgery physicians (*P* < .05 for all). Physicians with higher-volume venous ablation practices performed more EVTAs per patient compared with physicians with the lower-volume practices (≥35 EVTAs vs 11-18 EVTAs: AD, 0.63; 95% CI, 0.54-0.73; *P* < .001) (Table 3).

### Physician Performance in Response to Peer-Based Data

**Figure 1.**
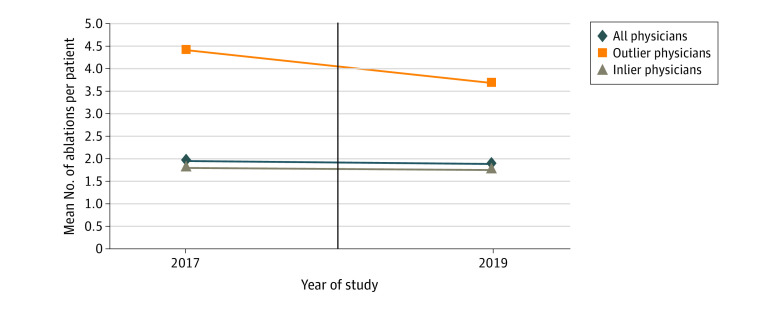
Mean Number of Ablations Performed per Patient by Physicians in 2017 vs 2019 Physicians who performed 3.4 or more endovenous thermal ablations per patient per year were considered outliers. Inlier and outlier status was based on physicians’ performance in 2017.

**Figure 2.**
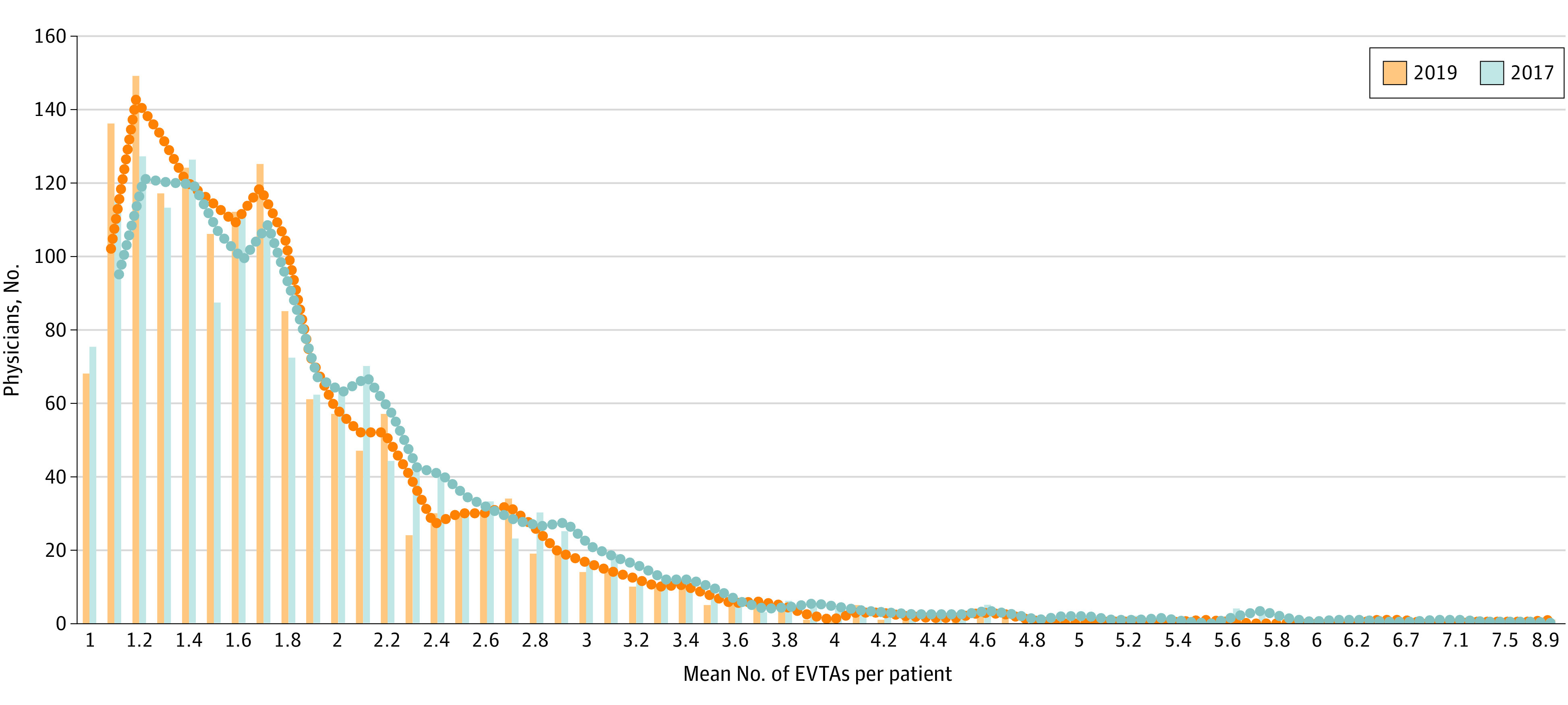
National Distribution of Physicians' Mean Number of Ablations per Patient in 2017 vs 2019 With Moving Mean Both the 2017 and 2019 groups included 1558 physicians. EVTAs indicates endovenous thermal ablations.

Among all physicians, the mean (SD) number of EVTAs performed per patient decreased from 2017 to 2019 (1.97 [0.85] vs 1.89 [0.77]; *P* < .001) (Figure 1). There was a modest decrease in the mean (SD) number of EVTAs per patient among inlier physicians (1.83 [0.57] vs 1.78 [0.55]; *P* < .001) and a more substantial decrease in the mean number of EVTAs per patient among outlier physicians (4.40 [1.01] vs 3.67 [1.41]; *P* < .001). Median values were similar (eFigure 1 in the Supplement). There was a leftward shift in the moving mean of EVTAs performed per patient in 2019 compared with 2017 (Figure 2).

Of the 1558 physicians included in the intervention, 90 (5.8%) performed 3.4 or more EVTAs per patient per year in 2017 and were originally classified as outliers. Of the 90 physicians classified as outliers, 71 (78.9%) reduced their EVTA use after the intervention. Based on 2019 EVTA use, 42 of those outlier physicians (46.7%) were reclassified as inlier physicians and 48 (53.3%) remained outlier physicians. In contrast, 16 of the 1468 physicians (1.1%) who were initially classified as inliers in 2017 were reclassified as outliers in 2019. The number of EVTAs performed per patient for each physician decreased by a mean (SD) of 0.09 (0.46) procedures overall (median, 0.10 procedures; IQR, −0.10 to 0.30 procedures; *P* < .001). The change in the median number of EVTAs was also evaluated (eFigure 2 in the Supplement).

### Sensitivity Analysis

We repeated our analysis including the new *CPT* codes for chemical ablation for the 2019 analysis. After addition of the new *CPT* codes, the mean (SD) number of EVTAs performed per patient increased from 1.93 (0.82) in 2017 to 2.04 (0.90) in 2019 (*P* < .001). However, this increase was mostly attributable to the inlier physicians (1.80 [0.56] in 2017 vs 1.93 [0.72] in 2019; *P* < .001). In contrast, the mean (SD) number of EVTAs performed by outliers persistently decreased from 2017 to 2019 even with the addition of the new codes (4.40 [1.01] in 2017 vs 4.03 [1.39] in 2019; *P* < .001) (eFigure 3 in the Supplement).

### Estimated Economic Impact

Among the 1558 physicians included in the pre- vs postintervention analysis, we identified a mean (SD) decrease of 0.09 (0.46) EVTAs per patient from 2017 to 2019. A total of 60 016 patients underwent a total of 127 209 EVTAs performed by 1558 physicians in 2019. The mean (SD) cost of each EVTA was $1179.18 ($377.96). The total Medicare-allowed cost for EVTA treatment in 2019 was $153 094 610. If the patients in 2019 had been treated with 0.09 more EVTAs per patient, the cost of EVTA treatment would have been $159 393 110, representing savings of $6.3 million to Medicare in 2019.

## Discussion

After analyzing EVTA use among Medicare billing prescribers in the US in 2017, we provided individualized peer-benchmarked report cards to all physicians in late 2018. In the present study, we found a change in the number of EVTAs performed per patient for both inlier and outlier physicians in 2019 compared with 2017. We found a significant reduction in EVTA use across the study period, particularly among outlier physicians. Of the original outlier physicians, 46.7% were reclassified as inlier physicians in the postintervention period, and the changes that we observed were not sensitive to the introduction of new technology or new billing codes. The reduction was associated with an estimated cost savings of $6.3 million to the Medicare system per year. Overall, the quality improvement initiative was associated with a significant reduction in waste related to the use of EVTA.

In a previous study describing EVTA practice patterns among physicians treating Medicare beneficiaries in 2017,^8^ a physician-developed quality metric for EVTA was applied and factors associated with level of use were analyzed. In that study, the mean number of EVTAs performed per patient per year was 1.9, but 106 physicians exceeded the mean by more than 2 SDs. In the present study, only 69 physicians met the 2017 definition of an outlier, and 46.7% of the original outlier physicians were reclassified as inliers. We also observed a significant decrease in the number of EVTAs performed per patient among outlier physicians. These data suggest that the peer-benchmarking initiative was associated with a substantial change in practice patterns in the use of EVTA. The precise mechanism by which the intervention may have incentivized change is probably multifactorial. The intervention, which was highlighted at the American Vein and Lymphatic Society 2018 Annual Meeting, alerted physicians that EVTA practice patterns were being scrutinized. Physicians who were performing more EVTAs per patient than their peers may have accessed resources or identified knowledge gaps, leading to a change in practice patterns. In a physician survey, fears of malpractice accusations or patient pressure were cited as top reasons for overtreatment in medicine,^14^ although financial incentives and lack of peer review and shared knowledge in private practice settings^15^ and lack of standard training, diagnostic protocols, and treatment guidelines may also play a role.^16,17^ Although we did not randomly assign physicians to receive or not receive the reports, the observation of a larger decrease in EVTA use among outlier physicians suggests that the feedback was informative to physicians practicing above the standard. However, 53.3% of outlier physicians remained outliers after peer benchmarking. Those physicians may have had more complex practices that necessitated increased use of EVTA compared with others, or they may have never obtained or read the report card that was sent to them. Future qualitative interviews will be important to understanding the reasons for and main barriers to change. Because this was an observational study, we could not assess whether the change in practice that we observed was causative. Although there was a temporal association between report distribution and practice change, it is unclear whether the reports or the potential knowledge of the reports were associated with changed behavior. A randomized clinical trial would be helpful to evaluate causation.

Examination of the entire cohort showed a reduction of 0.09 EVTAs per patient per year across the intervention period. This decrease in use of EVTAs, although clinically small, was associated with an estimated savings of $6.3 million in a single year. Given that the cost of doing the analysis and creating and distributing the reports was approximately $25 000, this finding demonstrates that small changes in individual EVTA practice patterns can translate into large absolute savings at the population level.

In our sensitivity analysis, there was an upward trend in the use of EVTAs in the overall cohort. Although we still observed a benefit associated with the performance feedback initiative among outlier physicians, the use of new technology for the treatment of chronic venous insufficiency represents an opportunity for new engagement. To maintain a long-term or additional effect, ongoing performance report distribution with repeated interventions will likely be needed.^18,19,20^

Peer benchmarking has been shown to be effective in other medical settings.^15,21,22,23^ For example, implementation of a similar quality improvement initiative using confidential individualized peer-benchmarking reports distributed to surgeons performing Mohs procedures was associated with an 83% decrease in the number of stages taken, with an estimated savings to Medicare of $11.1 million.^14^ Both the American College of Surgeons National Surgical Quality Improvement Initiative and the Society for Vascular Surgery Vascular Quality Initiative have used peer-benchmark scoring systems to provide institutions with feedback on outcomes of a wide variety of surgical metrics.^15,24,25,26^ Our study is unique, to our knowledge, in that we provided peer-benchmarked reports to individual physicians rather than institutions and observed a measurable and meaningful change in the use of a given procedure, rather than the outcome of the procedure. Like many quality improvement projects, the intervention will likely need ongoing performance report distribution with repeated interventions to maintain a long-term benefit.^18,19,20^

## Limitations

This study has limitations. First, the data were limited to Medicare-billed patient interactions, and therefore data from physicians who treat only non-Medicare patients were excluded. Although we could adjust for demographics and EVTA indication (ie, pain, swelling, and inflammation and ulceration), we could not analyze disease severity or clinical indications for individual patients. We were also unable to analyze the precise level of EVTA and were limited to sidedness and the information provided by the *CPT* code designations. Some patients may require variable care based on their clinical condition or response to prior therapy. This is consistent with our finding that patients with ulceration underwent more EVTAs than did patients with swelling, pain, or inflammation. Additional venous interventional procedures were introduced during the study period, and this may have introduced confounding into the analysis. However, we examined this closely in a sensitivity analysis that included the new codes, and the results still demonstrated a persistent significant reduction in EVTA use among outlier physicians regardless of which coding set was used.

## Conclusions

There is substantial variability in the use of EDTA performed per patient in the US.^8^ In this quality improvement study, we provided all physicians practicing EVTA with a 1-time peer-benchmarked performance report card. The timing of this intervention was associated with a statistically significant decrease in the number of EVTAs performed per patient. This change was greater among outlier physicians. Overall, the mean rate of EVTAs per patient decreased by 0.09 procedures each year. Although this was a modest decrease at the individual patient level, it translated into a considerable savings of $6.3 million by Medicare in 2019. The quality improvement intervention provides pilot evidence that peer measure data sharing may be a practical mechanism for quality improvement and cost reduction in health care.

## Notes

Supplement. **eTable.** *International Classification of Diseases, Tenth Revision, Clinical Modification* (*ICD-10-CM*) Diagnosis Codes of Venous Diseases **eFigure 1.** Example Peer Benchmarked Performance Reports Distributed to Inlier (Panel A) and Outlier (Panel B) Physicians Showing Their Endovenous Thermal Ablation Procedure Use per Patient in 2017 **eFigure 2.** Comparison of Median Number of Endovenous Thermal Ablation Procedures Performed per Patient by Physicians in 2017 vs 2019 **eFigure 3.** Comparison of Mean Number of Ablation Procedures Performed per Patient by Physicians in 2017 vs 2019 Including Expanded *CPT* Codes for Chemical Ablation Introduced in 2018 (Sensitivity Analysis)

## References

[1] Hamdan A. Management of varicose veins and venous insufficiency. JAMA. 2012;308(24):2612-2621. doi:10.1001/jama.2012.111352 23268520

[2] Piazza G. Varicose veins. Circulation. 2014;130(7):582-587. doi:10.1161/CIRCULATIONAHA.113.008331 25114187

[3] Pappas PJ, Lakhanpal S, Nguyen KQ, Fernandez E, Sufian S. Extended Center for Vein Restoration Study assessing comparative outcomes for the treatment of chronic venous insufficiency in Medicare- and non–Medicare-eligible patients. J Vasc Surg Venous Lymphat Disord. 2021;9(6):1426-1436.e2. doi:10.1016/j.jvsv.2021.04.015 33965612

[4] Davies AH. The seriousness of chronic venous disease: a review of real-world evidence. *Adv Ther*. 2019; 36(suppl 1):5–12. 10.1007/s12325-019-0881-7 PMC6824448 30758738

[5] Nesbit C, Eifell RK, Coyne P, Badri H, Bhattacharya V, Stansby G. Endovenous ablation (radiofrequency and laser) and foam sclerotherapy versus conventional surgery for great saphenous vein varices. Cochrane Database Syst Rev. 2011;5(10):CD005624. doi:10.1002/14651858.CD005624.pub2 21975750

[6] Pavlović MD, Schuller-Petrović S, Pichot O, . Guidelines of the First International Consensus Conference on Endovenous Thermal Ablation for Varicose Vein Disease—ETAV Consensus Meeting 2012. *Phlebology*. 2012;30(4):257–273. 10.1177/0268355514524568 24534341

[7] Prabhakar AM, Misono AS, Sheth RA, . Changing Medicare utilization of minimally invasive procedures for the treatment of chronic venous insufficiency. J Vasc Interv Radiol. 2017;28(6):818-824. doi:10.1016/j.jvir.2017.02.034 28396193

[8] Mann M, Wang P, Schul M, . Significant physician practice variability in the utilization of endovenous thermal ablation in the 2017 Medicare population. J Vasc Surg Venous Lymphat Disord. 2019;7(6):808-816.e1. doi:10.1016/j.jvsv.2019.06.019 31495766

[9] Research Data Assistance Center. Master beneficiary summary file base. Accessed July 13, 2021. https://resdac.org/cms-data/files/mbsf-base

[10] National Bureau of Economic Research. Census core-based statistical area to federal information processing series county crosswalk. Accessed July 13, 2021. https://www.nber.org/research/data/census-core-based-statistical-area-cbsa-federal-information-processing-series-fips-county-crosswalk

[11] Research Data Assistant Center. Medicare data on provider practice and specialty. Accessed July 13, 2021. https://resdac.org/cms-data/files/md-ppas

[12] Centers for Medicare & Medicaid Services. Explore & download Medicare provider data. Accessed July 13, 2021. https://data.cms.gov/provider-data/?redirect=true

[13] Brown GW, Mood AM. On median tests for linear hypotheses. Second Berkley Symposium. Accessed July 10, 2021. https://digitalassets.lib.berkeley.edu/math/ucb/text/math_s2_article-12.pdf

[14] Siah MC, Abramowitz SD, Haser P, Ricotta J, Woo EY, Macsata R. Evaluating the venous experience in vascular surgery training. J Vasc Surg Venous Lymphat Disord. 2017;5(3):446-452. doi:10.1016/j.jvsv.2017.01.015 28411714

[15] Albertini JG, Wang P, Fahim C, . Evaluation of a peer-to-peer data transparency intervention for Mohs micrographic surgery overuse. JAMA Dermatol. 2019;155(8):906-913. doi:10.1001/jamadermatol.2019.1259 31055597 PMC6503515

[16] Brownlee S, Chalkidou K, Doust J, . Evidence for overuse of medical services around the world. Lancet. 2017;390(10090):156-168. doi:10.1016/S0140-6736(16)32585-5 28077234 PMC5708862

[17] Lyu H, Xu T, Brotman D, . Overtreatment in the United States. PLoS One. 2017;12(9):e0181970. doi:10.1371/journal.pone.0181970 28877170 PMC5587107

[18] Leeds IL, Wick EC. *Surgical quality improvement: local quality improvement*. In: Kelz R, Wong S, eds. *Surgical Quality Improvement. Success in Academic Surgery*. Springer; 2017:45-53. doi:10.1007/978-3-319-23356-7_5

[19] Stonko DP, O Neill DC, Dennis BM, Smith M, Gray J, Guillamondegui OD. Trauma quality improvement: reducing triage errors by automating the level assignment process. J Surg Educ. 2018;75(6):1551-1557. doi:10.1016/j.jsurg.2018.03.014 29656835

[20] Anthony SG, Prevedello LM, Damiano MM, . Impact of a 4-year quality improvement initiative to improve communication of critical imaging test results. Radiology. 2011;259(3):802-807. doi:10.1148/radiol.11101396 21467253

[21] Kaczmarski K, Wang P, Gilmore R, . Surgeon re-excision rates after breast-conserving surgery: a measure of low-value care. J Am Coll Surg. 2019;228(4):504-512.e2. doi:10.1016/j.jamcollsurg.2018.12.043 30703538

[22] Hicks CW, Liu J, Yang WW, . A comprehensive Choosing Wisely quality improvement initiative reduces unnecessary transfusions in an academic department of surgery. Am J Surg. 2017;214(4):571-576. doi:10.1016/j.amjsurg.2017.06.020 28683893

[23] Sacarny A, Barnett ML, Le J, Tetkoski F, Yokum D, Agrawal S. Effect of peer comparison letters for high-volume primary care prescribers of quetiapine in older and disabled adults: a randomized clinical trial. JAMA Psychiatry. 2018;75(10):1003-1011. doi:10.1001/jamapsychiatry.2018.1867 30073273 PMC6233799

[24] Edenfield L, Blazick E, Eldrup-Jorgensen J, . Outcomes of carotid endarterectomy in the Vascular Quality Initiative based on patch type. J Vasc Surg. 2020;71(4) 1260–1267. doi:10.1016/j.jvs.2019.05.063 31492613

[25] Vollmer CM Jr, Lewis RS, Hall BL, . Establishing a quantitative benchmark for morbidity in pancreatoduodenectomy using ACS-NSQIP, the Accordion Severity Grading System, and the Postoperative Morbidity Index. Ann Surg. 2015;261(3):527-536. doi:10.1097/SLA.0000000000000843 25268299

[26] Lewis CM, Aloia TA, Shi W, . Development and feasibility of a specialty-specific national surgical quality improvement program (NSQIP): the head and neck–reconstructive surgery NSQIP. JAMA Otolaryngol Head Neck Surg. 2016;142(4):321-327. doi:10.1001/jamaoto.2015.3608 26892756 PMC5859938

