# Incorrect Spelling of a Surname in the Byline

**DOI:** 10.1001/jamanetworkopen.2021.47752

**Published:** 2022-01-18

In the Original Investigation titled “Evaluation of a Physician Peer-Benchmarking Intervention for Practice Variability and Costs for Endovenous Thermal Ablation,”^1^ published December 14, 2021, the surname of Marlin Schul, MD, MBA, was spelled incorrectly. This article has been corrected.^1^
