# Supplementary material for: Evaluation of a Physician Peer-Benchmarking Intervention for Practice Variability and Costs for Endovenous Thermal Ablation
Source: JAMA Netw Open. 2021 Dec 14;4(12):e2137515. doi: 10.1001/jamanetworkopen.2021.37515 (PMC8672233; doi:10.1001/jamanetworkopen.2021.37515)
Supplement: Supplement. — eTable. International Classification of Diseases, Tenth Revision, Clinical Modification (ICD-10-CM) Diagnosis Codes of Venous Diseases eFigure 1. Example Peer Benchmarked Performance Reports Distributed to Inlier (Panel A) and Outlier (Panel B) Physicians Showing Their Endovenous Thermal Ablation Procedure Use per Patient in 2017 eFigure 2. Comparison of Median Number of Endovenous Thermal Ablation Procedures Performed per Patient by Physicians in 2017 vs 2019 eFigure 3. Comparison of Mean Number of Ablation Procedures Performed per Patient by Physicians in 2017 vs 2019 Including Expanded CPT Codes for Chemical Ablation Introduced in 2018 (Sensitivity Analysis) [file jamanetwopen-e2137515-s001.pdf]

Supplementary Online Content

Stonko DP, Dun C, Walsh C, et al. Evaluation of a physician peer-benchmarking intervention for practice variability and costs for endovenous thermal ablation. *JAMA Netw Open*. 2021;4(12):e2137515. doi:10.1001/jamanetworkopen.2021.37515

eTable. *International Classification of Diseases, Tenth Revision, Clinical Modification* (*ICD-10-CM*) Diagnosis Codes of Venous Diseases

eFigure 1. Example Peer Benchmarked Performance Reports Distributed to Inlier (Panel A) and Outlier (Panel B) Physicians Showing Their Endovenous Thermal Ablation Procedure Use per Patient in 2017

eFigure 2. Comparison of Median Number of Endovenous Thermal Ablation Procedures Performed per Patient by Physicians in 2017 vs. 2019

eFigure 3. Comparison of Mean Number of Ablation Procedures Performed per Patient by Physicians in 2017 vs. 2019 Including Expanded CPT Codes for Chemical Ablation Introduced in 2018 (Sensitivity Analysis)

This supplementary material has been provided by the authors to give readers additional information about their work.

© 2021 Stonko DP et al. *JAMA Network Open*.

eTable. ***International Classification of Diseases, Tenth Revision, Clinical Modification*** **(*ICD-10-CM*) diagnosis codes of venous diseases.**

**Category**	***ICD-10-CM* diagnosis codes**
**Pain/swelling/inflammation:**	I8000, I8001, I8002, I8003, I80209, I80291, I803, I8310, I8311, I8312, I83811, I83812, I83813, I83819, I83891, I83892, I83893, I83899, I87001, I87002, I87003, I87021, I87022, I87023, I87091, I87092, I87093, I872, I87301, I87302, I87303, I87309, I87321, I87322, I87323, I87329, I87391, I87392, I87393, I87399, I878, I879, M79604, M79605, M79606, M79609, M79651, M79661, M79662, M79669, R601, R609, R252
**Ulceration:**	I83001, I83002, I83003, I83004, I83005, I83008, I83011, I83012, I83013, I83014, I83015, I83018, I83021, I83022, I83023, I83024, I83025, I83028, I83201, I83202, I83203, I83204, I83205, I83208, I83209, I83211, I83212, I83213, I83214, I83215, I83218, I83219, I83221, I83222, I83223, I83224, I83225, I83228, I83229, I87011, I87012, I87013, I87031, I87032, I87033, I87311, I87312, I87313, I87319, I87331, I87332, I87333, I83009, I83019, I83029, I83209, I83229, L97211, L97219, L97309, L97311, L97321, L97329, L97811, L97819, L97821, L97909, L97919, L97921, L97929, S81809A

© 2021 Stonko DP et al. *JAMA Network Open*.

eFigure 1. Example peer benchmarked performance reports distributed to inlier (panel A) and outlier (panel B) physicians showing their endovenous thermal ablation procedure use per patient in 2017.

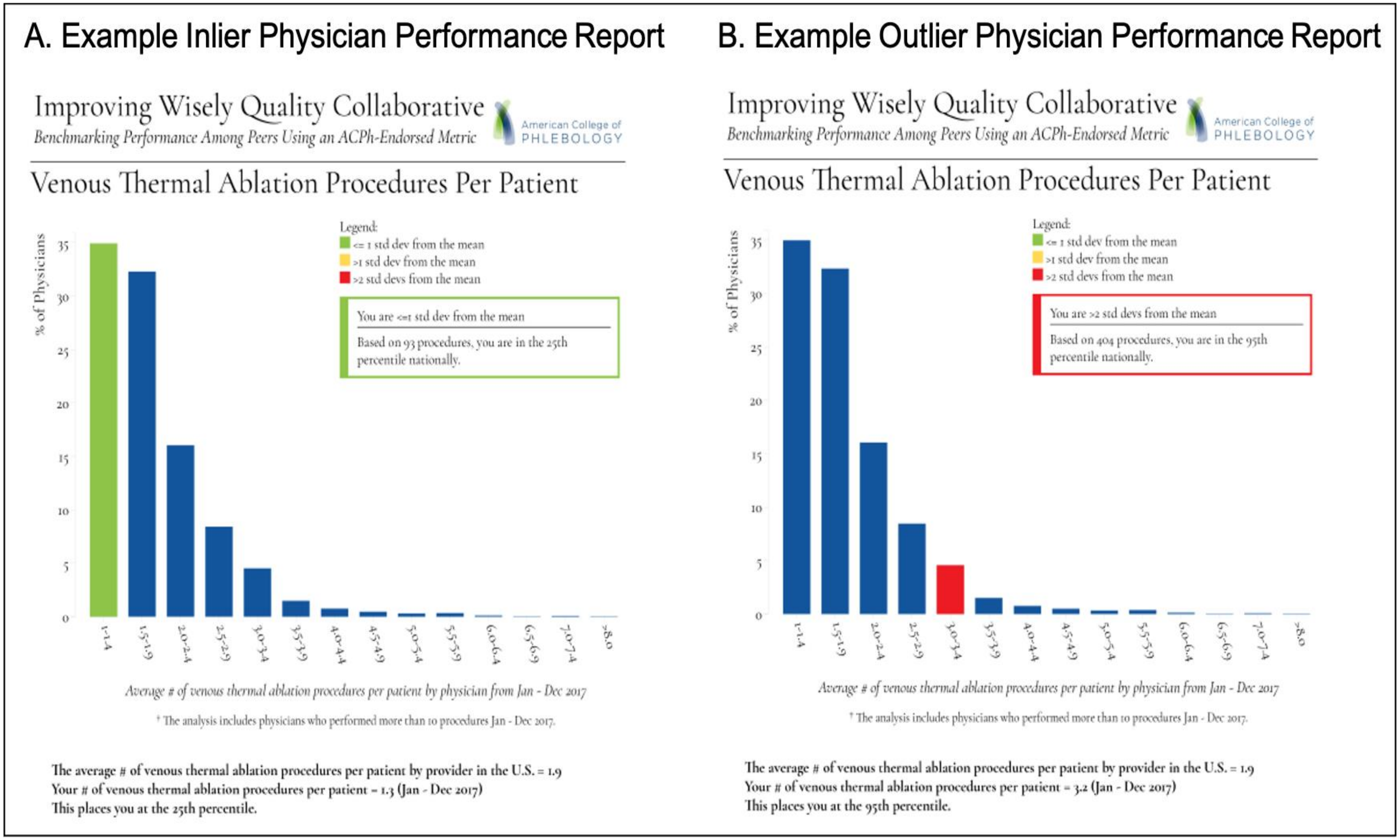

© 2021 Stonko DP et al. *JAMA Network Open*.

eFigure 2. Comparison of median number of endovenous thermal ablation procedures performed per patient by physicians in 2017 vs. 2019

**Inlier and outlier status was based on physicians' performance in 2017*

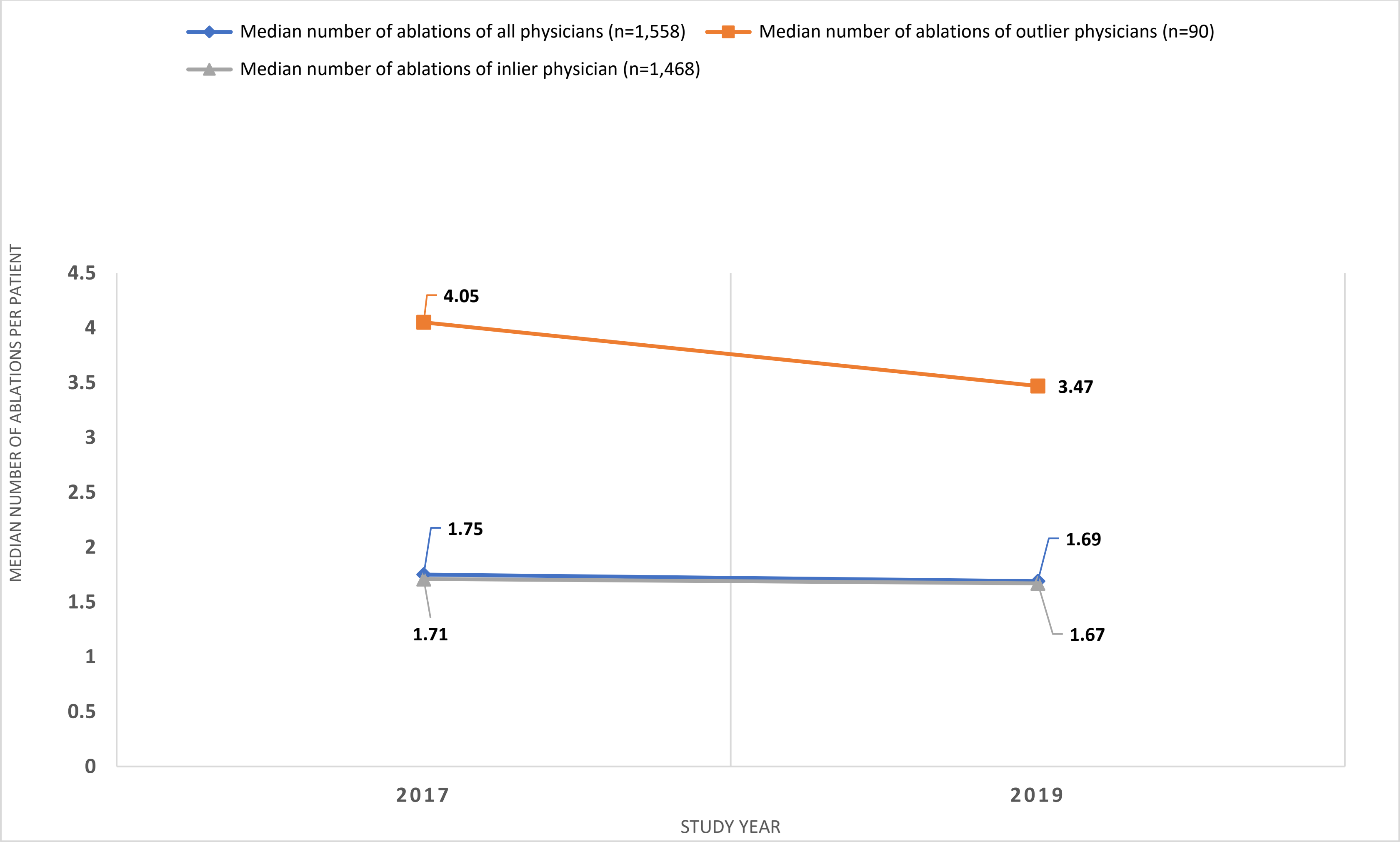

© 2021 Stonko DP et al. *JAMA Network Open*.

eFigure 3. Comparison of mean number of ablation procedures performed per patient by physicians in 2017 vs. 2019 including expanded CPT codes for chemical ablation introduced in 2018 (sensitivity analysis).

**Inlier and outlier status was based on physicians' performance in 2017*

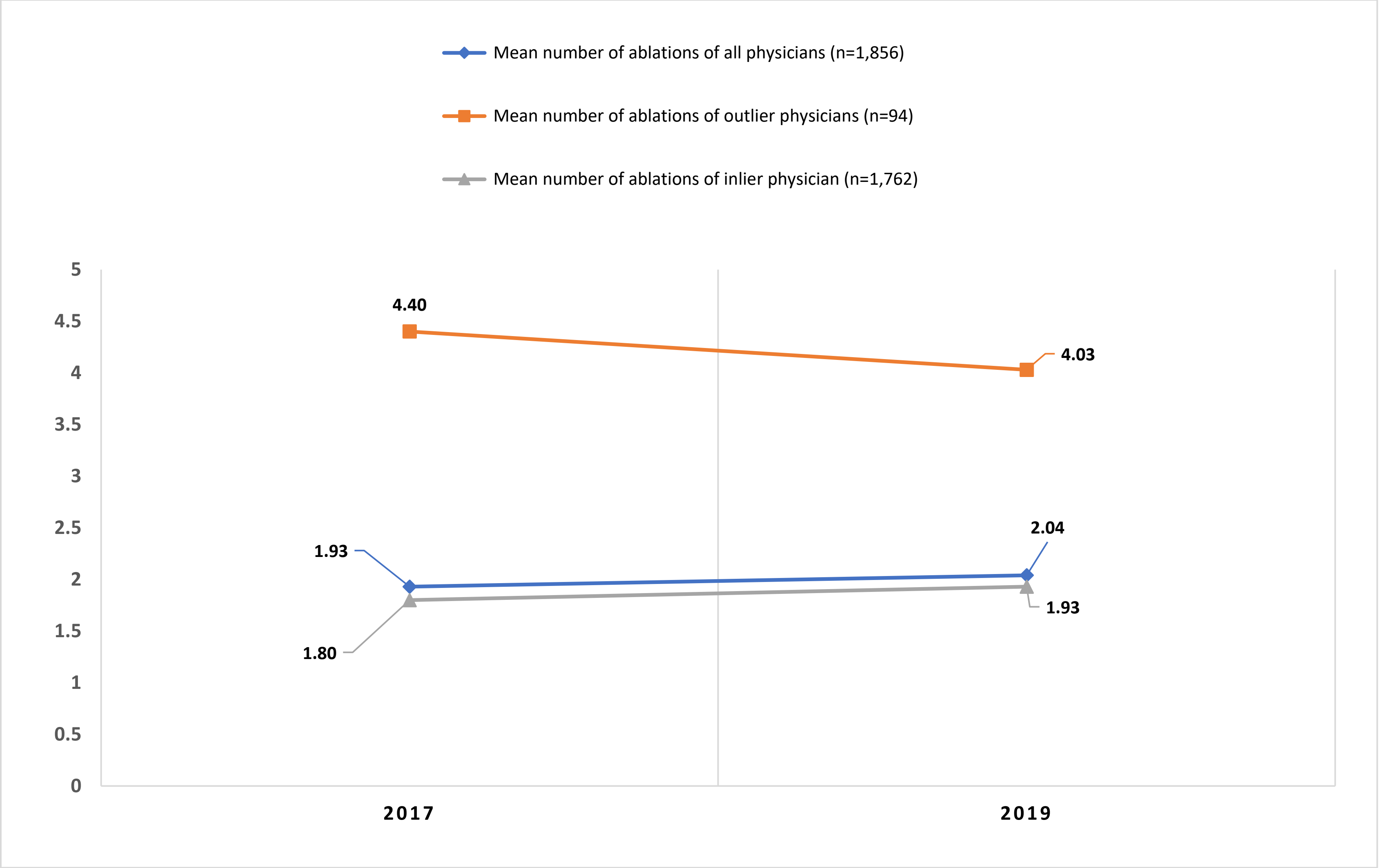

© 2021 Stonko DP et al. *JAMA Network Open*.
